# Efficacy of a simplified photobiomodulation protocol in orthodontic patients: a randomized crossover clinical trial

**DOI:** 10.1590/2177-6709.31.1.e2625100.oar

**Published:** 2026-04-17

**Authors:** Mylka Suellen de Moura Barros SANTOS, Renata Travassos da Rosa Moreira BASTOS, Nathália Carolina Fernandes FAGUNDES, David NORMANDO

**Affiliations:** 1 Federal University of Pará, Dental School (Belém/PA, Brazil).; 2 University Center of Pará, Dental School (Belém/PA, Brazil).; 3 University of Alberta, Dental School (Edmonton, Canada).; 4 Federal University of Pará, Dental School, Department of Orthodontics (Belém/PA, Brazil).

**Keywords:** Low-level light therapy, Orthodontics, Pain management, Terapia com luz de baixa intensidade, Ortodontia, Manejo da dor

## Abstract

**Introduction:**

Pain in orthodontic treatments results from an inflammatory process due to the application of forces. Despite photobiomodulation having shown effectiveness in reducing pain sensitivity, the method requires multiple sessions, which complicates its practical application in orthodontic clinics.

**Objective:**

To test a simplified protocol of photobiomodulation with a single application in adults undergoing orthodontic treatment.

**Material and methods:**

This single-blind, randomized, crossover study evaluated 30 participants undergoing fixed orthodontic treatment. Half of them received a single application of photobiomodulation, while the other half received a placebo, with group crossover after one month. The application was performed at 10 points using a GaAlAs diode laser (100 mW/cm^2^) immediately after appliance activation. Pain was assessed using a visual analog scale (VAS) at T0 (before the orthodontic check-up appointment), T24h (24 hours after the orthodontic control appointment), and T48h (48 hours after the orthodontic control appointment), and data were analyzed using two-way repeated measures ANOVA (time and intervention).

**Results:**

A total of 27 participants were evaluated. Pain was significantly higher at T24h than at T48h in both groups. Although there was no difference in pain sensitivity between the groups at T24h, pain significantly decreased in the intervention group after 48 hours. The variables of sex and age did not influence pain perception.

**Conclusion:**

The study shows that photobiomodulation can help control orthodontic pain, being well accepted by participants and not extending clinical time. However, the analgesic effects are still limited, indicating the need to refine the protocol to enhance its effectiveness.

## RESUMO

**Introdução:**

A dor nos tratamentos ortodônticos decorre de um processo inflamatório desencadeado pela aplicação de forças. Embora a fotobiomodulação tenha demonstrado eficácia na redução da sensibilidade dolorosa, o método geralmente requer múltiplas sessões, o que dificulta sua aplicação prática na rotina clínica ortodôntica.

**Objetivo:**

Testar um protocolo simplificado de fotobiomodulação, com aplicação única, em adultos submetidos a tratamento ortodôntico.

**Material e Métodos:**

Este estudo randomizado, simples-cego, cruzado, avaliou 30 participantes em tratamento ortodôntico com aparelho fixo. Metade dos participantes recebeu aplicação única de fotobiomodulação, enquanto a outra metade recebeu placebo, ocorrendo o cruzamento dos grupos após um mês. A aplicação foi realizada em 10 pontos, utilizando laser diodo GaAlAs (100 mW/cm^2^), imediatamente após a ativação do aparelho. A dor foi avaliada por meio da escala visual analógica (EVA) nos tempos T0 (antes da consulta de controle ortodôntico), T24h (24 horas após a consulta de controle ortodôntico) e T48h (48 horas após a consulta de controle ortodôntico), e os dados foram analisados por meio de ANOVA de medidas repetidas em dois fatores (tempo e intervenção).

**Resultados:**

Foram avaliados 27 participantes. A dor foi significativamente maior em T24h em comparação a T48h, em ambos os grupos. Embora não tenha havido diferença na sensibilidade dolorosa entre os grupos em T24h, observou-se redução significativa da dor no grupo intervenção após 48 horas.

**Conclusão:**

O estudo demonstra que a fotobiomodulação pode auxiliar no controle da dor ortodôntica. Contudo, os efeitos analgésicos ainda são limitados, indicando a necessidade de aprimoramento do protocolo para potencializar sua eficácia.

## INTRODUCTION

Pain is a major reason for discontinuing orthodontic treatment, as it affects patients’ daily activities, mood, and overall health, leading to fear and reduced adherence to treatment.^1^ Being a subjective response, pain exhibits wide interindividual variability and may be influenced by age, pain threshold, force intensity, emotional and stress levels, cultural background, previous experiences, and, in women, the menstrual cycle.^2^

Orthodontic pain is associated with real or potential injury to periodontal tissues, resulting from inflammatory processes related to bone remodeling, reorganization of periodontal ligament fibers, bone resorption and formation, and vascular changes.^3^ Additionally, mechanical forces from orthodontic appliances activate nerve endings, contributing to nociceptive responses.

Various strategies have been proposed for pain management, including the use of analgesics and anti-inflammatory drugs,^4^ ^,^ ^5^ chewing gum,^6^ acupuncture and acupressure,^7^ and laser therapy (photobiomodulation).^1^ ^,^ ^8^ ^-^ ^12^ Photobiomodulation consists of applying low-intensity light to oral tissues, such as gingiva and alveolar bone, to modulate cellular activity and reduce discomfort during tooth movement.^13^ Evidence indicates that this approach decreases peripheral nerve sensitivity, significantly reducing pain intensity and duration associated with orthodontic procedures.^8^ ^-^ ^10^ ^,^ ^14^ Reported reductions reach approximately 24%, with additional benefits such as safety, absence of side effects, and improved patient comfort.^12^ ^,^ ^15^ ^-^ ^19^

Despite its efficacy, the most commonly used protocols involve multiple sessions throughout treatment, which limits its routine clinical applicability. Thus, evaluating the effectiveness of a simplified protocol, based on a single application, is clinically relevant to facilitate implementation, improve adherence, and optimize patient comfort while reducing chair time and operational demands.

## MATERIAL AND METHODS

### REGISTRATION

This single-blind, randomized, controlled crossover study was registered in the Brazilian Registry of Clinical Trials (ReBEC) under no. RBR-5v35crn and reported following CONSORT guidelines for crossover trials.^20^

### CONSENT

The study followed the guidelines for Research Involving Human Subjects (Resolution 466/12 of the CNS)^21^ and was approved by the Research Ethics Committee of the Health Sciences Institute, Federal University of Pará (No. 6.504.742). Patient authorization was obtained through the Informed Consent Form and Assent Form, signed by the guardians of those under 18 years of age.

### SAMPLE SIZE CALCULATION

Sample size was calculated using G*Power (version 3.1.9.4, Düsseldorf, Germany)^22^ for paired mean comparisons, assuming a one-unit difference in the visual analog scale (VAS), 1.7^9^standard deviation, 0.8 power, and 0.05 alpha to assess the reduction of pain from moderate to mild, assuming this as a clinically significant difference on the VAS following photobiomodulation after orthodontic adjustment. A sample of 25 was estimated, with 30 participants (15 males, 15 females) required to account for 20% loss.

### ELIGIBILITY CRITERIA

Participants were recruited from dental clinics affiliated with four orthodontic specialization programs and two private dental clinics, all located in the city of Belém, Pará, Brazil.

### INCLUSION CRITERIA

Participants aged between 12 and 50 years, of both sexes, in the initial alignment and leveling phase of orthodontic treatment were included. Treatment was performed without extractions, using preadjusted brackets with Roth prescription and a 0.022 × 0.028-in slot (Morelli, Sorocaba, Brazil). Participants were considered to be in the alignment and leveling phase if they were using NiTi archwires ranging from 0.012-in to 0.020-in.

### EXCLUSION CRITERIA

Participants were excluded if they had chronic pain; systemic, periodontal, or periapical diseases; a history of previous orthodontic treatment; were pregnant or breastfeeding; had facial neuralgia or psychological disorders; were using analgesics, anti-inflammatory drugs, anticonvulsants, steroids, or bisphosphonates on a continuous basis; or presented with poor oral hygiene, defined as clinically evident dental plaque accumulation, calculus, and gingivitis. Additionally, participants were excluded if they did not attend the second appointment within a maximum interval of two months from the first appointment, or if they did not complete the VAS correctly.

### GROUP DESCRIPTION

At the initial orthodontic appliance activation appointment, participants were divided into two groups: a control group, (did not receive photobiomodulation), and an intervention group, (received photobiomodulation). At the following month’s appointment (after a one-month washout period), the group allocation was reversed.

### RANDOMIZATION

Randomization used opaque envelopes with a 1:1 allocation ratio. Sealed envelopes were prepared before the study, and participants randomly selected one. The examiner opened the envelope to assign the participant to a group. 

An envelope marked “0” assigned the participant to the control group, with no photobiomodulation at the first appointment. An envelope marked “1” assigned them to the intervention group, receiving photobiomodulation. After one month, group assignments were reversed.

### BLINDING

Participants were blinded to both the randomization and the intervention. It was not possible to blind the operator.

### OPERATOR CALIBRATION

The calibration of the operator who administered the laser application involved ensuring that the qualified professional followed the established instructions and protocols accurately. The operator used the instruction manual for the Laser Duo device, manufactured by MMOptics (São Carlos, SP, Brazil), ensuring adherence to the technical guidelines. Furthermore, the study protocol was applied precisely, with all results documented in detail to maintain compliance with the scientific and clinical standards required for correct laser therapy application.

### STUDY EXECUTION

**Figure 1:**
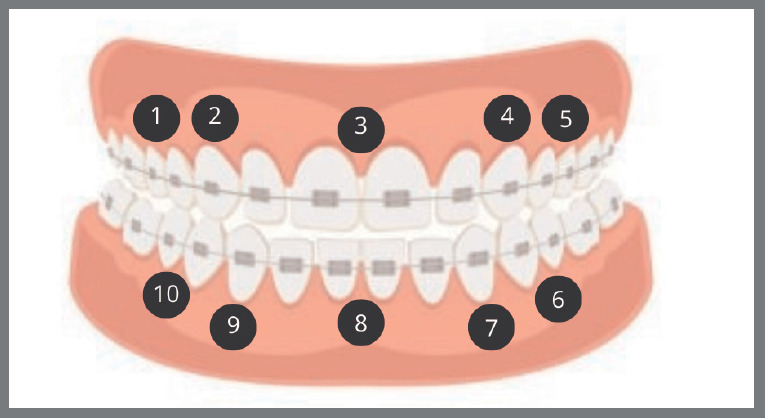
Laser application points: **1)** between the upper right premolars; **2)** upper right canine; **3)** upper midline; **4)** upper left canine; **5)** between the upper left premolars; **6)** between the lower left premolars; **7)** lower left canine; **8)** lower midline; **9)** lower right canine; **10)** between the lower right premolars.

After orthodontic appliance activation, intervention group participants received a single photobiomodulation session following a predefined protocol. A GaAlAs diode laser (808 nm wavelength, 3 J/cm^2^ energy, 100 mW/cm^2^ power density) was applied for 5 minutes to 10 points, being 5 on the upper arch and 5 on the lower arch (Fig. 1), targeting the cervical-vestibular root área.^10^ A LASER DUO MMOptics device (São Carlos, SP, Brazil) was used in L2 (infrared) mode with a proximity tip.

During the control session, the laser device remained off, and a pre-recorded laser sound played for 5 minutes, simulating photobiomodulation. Control parameters were set to 0 mW/cm^2^, 0 nm, and 0 J/cm^2^. 

To prevent carryover effects, a 30-day washout period was applied,^23^ given the single photobiomodulation application and the short-term nature of orthodontic pain.^24^ Vector images were created with Canva^®^ AI, and the translation to American English was performed using ChatGPT 4.0^®^.

### DATA COLLECTION

Participants were instructed in person to complete the VAS at T0 (before the appointment), T24h (24 hours post-photobiomodulation), and T48h (48 hours post-photobiomodulation). They recorded pain levels at home and returned the VAS sheet at the next appointment. Pain was measured using a 10-cm ruler, with results recorded in Excel^®^ (Redmond, WA, USA). 

At the second appointment, participants evaluated treatment discontinuation likelihood and the value of a 5-minute photobiomodulation extension, via WhatsApp^®^ surveys. Questions included: 1) “Would your current pain make you consider stopping treatment? Yes or No?” and 2) “On a scale of 0 to 10, rate the value of adding 5 minutes for laser application, based on pain relief.” 

Participants failing to complete the VAS correctly or missing the second appointment within two months were excluded. 

Primary outcome: intensity of orthodontic pain post-activation. Secondary outcomes: a) treatment discontinuation likelihood due to pain; b) value of a 5-minute photobiomodulation extension; c) influence of gender and age on pain perception.

### MEASUREMENT ERROR

The VAS at T24h for all participants was measured immediately after data collection and again 15 days later, to assess measurement error using intraclass correlation analysis. Intraclass correlation (ICC) was used to evaluate agreement between measurements taken at different times.

### STATISTICAL ANALYSIS

Descriptive statistics were performed for sex, age, and treatment discontinuation/adherence due to pain perception. Data normality was assessed with the Shapiro-Wilk test for T0, T24h, and T48h. Repeated-measures ANOVA analyzed sex and age interactions with pain perception, using Bonferroni correction for multiple comparisons. Analyses were conducted in Jamovi (version 1.6.18, Sydney, Australia) at a 5% significance level.

## RESULTS

The 30 participants, aged 12 to 50, were recruited from six orthodontic clinics between December 15, 2023, and March 28, 2024, with equal gender distribution (15 males and 15 females) and a mean age of 22.7 ± 10.8 years for females and 22.7 ± 8.7 years for males.

**Figure 2:**
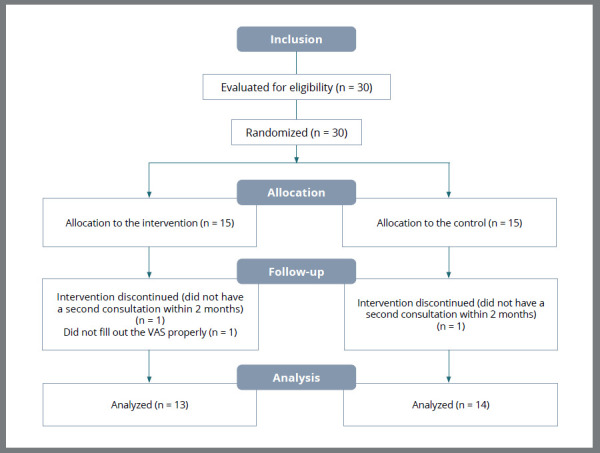
Participant recruitment and allocation flowchart.

Three participants were excluded: two for not attending the second visit within two months, and one for incorrectly filling out the VAS. Thus, 27 participants remained for the final data analysis (Fig. 2).

### MEASUREMENT ERROR ANALYSIS

Intraclass correlation coefficient (ICC) was used to assess reliability between repeated T24h VAS measurements in control and intervention sessions. This time point was chosen as most readings were above zero. ICC analysis on VAS scores at T24h, repeated after 15 days, yielded ICC = 0.99, indicating high measurement reliability. 

### PAIN PERCEPTION IN CONTROL AND INTERVENTION GROUPS OVER TIME

Post-activation pain significantly increased at 24 and 48 hours in both groups, compared to T0 (p<0.001). However, no difference was found between the control and intervention groups at any point (p=0.93). Gender (p=0.18) and age (p=0.23) did not influence orthodontic pain perception (Table 1).

**Table 1:** ANOVA on the effectiveness of photobiomodulation comparing the control and intervention groups over time, as well as the influence of sex and age.

Variables	F	p-value
Groups	0.007	0.93
Times	17.84	< .001
Groups * Times	0.95	0.39
Sex	1.83	0.18
Age	1.51	0.023

**Figure 3:**
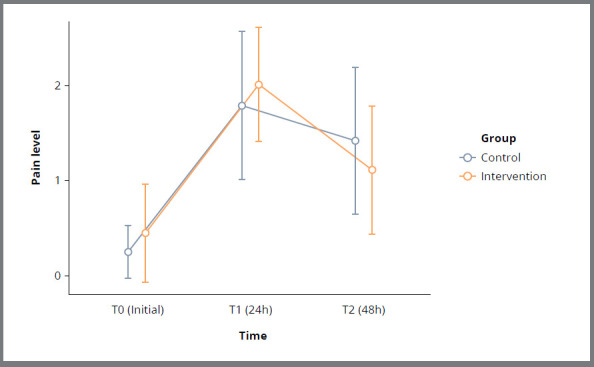
Mean and 95% CI for pain perception in control and intervention sessions at T0, T24h, and T48h.

Within each group, pain significantly increased in the first 24 hours, then significantly decreased between 24 and 48 hours (p < 0.001, Table 1, Fig. 3). Both groups showed an approximate 2-point increase in pain on a 0-10 scale within the first 24 hours. Between 24 and 48 hours, pain reduction was not significant in the control group (-0.35, p=0.26), but was significant in the intervention group (-0.89, p=0.007, Table 2).

**Table 2:** Mean, 95% confidence interval ( 95%CI ), difference in means and p-value (Bonferroni ) of pain perception in the control and intervention consultations at T0, T24h and T48h.

Group	Time	Mean	95% CI	Difference in means	p-value
			Lower - Upper		
Control	T0 (initial)	0.25	-0.02 - 0.53		
	T24h	1.78	1.01 - 2.56	T24h - T0 = 1.51	0.001**
	T48h	1.42	0.64 - 2.19	T48h - T24h = -0.35	0.26
Intervention	T0 (initial)	0.44	-0.06 - 0.95		
	T24h	2.00	1.41 - 2.60	T24h - T0 = 1.54	<0.001**
	T48h	1.11	0.44 - 1.78	T48h - T24h = -0.89	0.007*

* Alpha value adjusted by Bonferroni correction.

** Alpha value.

### CARRYOVER EFFECT

To evaluate pain perception, comparisons were made between intervention and control treatments, as well as across T0, T24h, and T48h using repeated-measures ANOVA (Table 3). Additionally, a 30-day washout between control and intervention treatments effectively prevented any residual laser effects during the control session evaluation. No statistically significant differences were found for T24h control vs. T24h intervention (p=0.67) and T48h control vs. T48h intervention (p=0.50), indicating no carryover effect (Table 3).

**Table 3:** ANOVA with Bonferroni correction comparing the Control and Intervention groups at times T0, 24 (T24h ) and 48 hours (T48h ).

Post Hoc Comparisons - Groups X Times						
Comparison					Difference in means	p
Groups	Times		Groups	Times		
Control	T0 (Initial)	-	Control	T24h	1.51	< .001**
Control	T24h	-	Control	T48h	-0.35	0.26
Intervention	T48h	-	Intervention	T24h	1.54	< 0.001**
Intervention	T0 (Initial)	-	Intervention	T48h	-0.89	0.007*
Control	T24h	-	Intervention	T0	0.18	0.50
Control	T48h	-	Intervention	T24h	0.22	0.67
Control	T48h	-	Intervention	T48h	- 0.31	0.50

* Adjusted alpha = 0.0071 (Bonferroni).

** Alpha value.

## QUESTIONNAIRE RESPONSES

### PERCEIVED IMPORTANCE OF PHOTOBIOMODULATION DESPITE EXTENDED APPOINTMENT TIME

Participants rated the importance of adding photobiomodulation to monthly orthodontic appointments, extending the session by 5 minutes, with a median score of 9, although responses varied widely from 1 to 10.

### TREATMENT ABANDONMENT DUE TO PAIN PERCEPTION AFTER INTERVENTION

Only two participants (7%) indicated that the pain experienced after the intervention would be sufficient reason to abandon orthodontic treatment, suggesting photobiomodulation was not effective enough to alleviate pain for these individuals (Table 4).

**Table 4:** Assessment of the possibility of withdrawal/abandonment of treatment in the face of self-perceived orthodontic pain after application of photobiomodulation carried out with a binomial proportion test.

Question	Answer	F (%)	Total	p
Would the level of pain you feel today be a determining factor in giving up/abandoning treatment?	No	25 (92%)	27	< 0.001
	Yes	2 (7%)	27	

### ADVERSE EVENTS

No adverse events were observed.

## DISCUSSION

Although the present findings indicate the potential of photobiomodulation (PBM) for orthodontic pain control, variability in application parameters may affect its effectiveness. Several studies have reported a reduction in pain perception - modulated by multiple individual factors^10^ - in patients receiving PBM, suggesting that this approach can improve comfort, by reducing peripheral nerve sensitivity and decreasing both pain intensity and duration.^8^ ^-^ ^10^ ^,^ ^13^ ^-^ ^19^ However, the absence of standardized protocols limits comparability across studies, as parameters and application frequencies differ considerably.^8^ Thus, optimizing the number of sessions and defining clinically feasible protocols is essential, since multiple applications restricted to office settings remain a challenge.^16^

In this study, monthly application did not significantly alter the typical cycle of orthodontic pain onset, peak, and resolution,^12^ but had limited effect on intensity, in line with systematic review data^16^ indicating the need for reapplication within 24 hours to achieve consistent analgesia. Regarding laser parameters, the most effective wavelengths for pain reduction range from 632 to 1,964 nm, with 800-830 nm being widely used for their analgesic efficacy.^14^ ^,^ ^23^ A systematic review^16^ identified 810 nm as ideal, and the present study used 808 nm, close to this range. Nevertheless, analgesic outcomes are not determined solely by wavelength, since other variables also influence PBM effectiveness.^8^

Systematic reviews commonly highlight the GaAlAs diode as the most effective for orthodontic pain management,^9^ ^,^ ^10^supporting its use in this study. Its near-infrared emission (800-900 nm) penetrates deeply, is absorbed by mitochondria, stimulates ATP synthesis, and promotes repair and analgesia.^8^ ^,^ ^16^ In addition, energy density is a critical parameter, with the ideal range being 0.05-10 J/cm^2^, as higher doses may be inhibitory.^11^ The 3 J/cm^2^ applied here aligns with recommendations to balance efficacy and safety.^19^ Other factors, including application frequency, emission mode, irradiation style, power, and exposure time, still require standardization.^10^ While studies with 2-3 day intervals demonstrated greater analgesia,^24^ they demand repeated visits, limiting feasibility. A single dose may reduce pain to tolerable levels,^16^ but in the present study its impact was restricted to the second day after activation.

Greater analgesic effects have been reported with multiple application points,^14^ ^,^ ^24^ which extend clinical time to over 5 minutes and require 2-3 sessions.^24^ To maintain feasibility, the present protocol was limited to 5 minutes, consistent with other clinical trials showing significant reduction within this timeframe.^14^ ^,^ ^23^ However, unlike previous reports,^14^ ^,^ ^24^ the simplified protocol did not alter peak pain intensity or its temporal course. This reinforces the need to improve protocols by increasing the number of application points^14^ ^,^ ^24^ and covering all teeth involved.^16^

Overall, these results suggest that single-session PBM has limited analgesic potential, as it does not adequately address the inflammatory and neuronal processes underlying orthodontic pain, which peak within the first 24-72 hours after appliance activation. In contrast, multi-session protocols provide repeated stimuli, sustaining anti-inflammatory and regenerative effects and accounting for the superior outcomes described in the literature.^25^

Despite modest analgesic efficacy, most participants valued PBM during monthly visits, with 77.7% reporting it as important and 92% indicating no intention to discontinue treatment due to pain. This highlights its role within a humanized, patient-centered approach, consistent with reports that patients often value such supportive measures more than clinicians.^26^ Thus, even with limited clinical effect, PBM may strengthen the patient-clinician relationship and enhance adherence.

Finally, no significant differences in pain perception were observed regarding sex or age, consistent with part of the literature.^27^ Nonetheless, some studies report greater pain sensitivity in women, possibly related to hormonal factors,^28^ and higher pain in adults compared with younger patients.^24^ Others indicate that adolescents, particularly females aged 13-16, report pain more frequently and with greater impact.^3^ These findings reinforce the multifactorial nature of orthodontic pain perception and the need for individualized approaches.

## LIMITATIONS

This study had several limitations, including reliance on participants’ accurate understanding of orthodontic pain perception,^1^ ^,^ ^15^ ^,^ ^29^ missed monthly appointments, and the initial aim to minimize interference in orthodontic sessions by limiting photobiomodulation application to 5 minutes.^10^ ^,^ ^19^

Another limitation was the absence of a control group using a conventional photobiomodulation system,^8^ ^,^ ^16^ which would allow for a broader assessment of the simplified protocol’s efficacy.

Managing the carryover effect is essential in crossover trials and presented a limitation here, as treatments had to be administered so that one would not interfere with the other, maintaining result validity.^30^

Further detailed studies are needed, applying this protocol to a more specific population,^14^ ^,^ ^19^ ^,^ ^24^ with longer follow-up periods,^3^ and using a greater number of application points.^10^

## CONCLUSION

The findings of this study reinforce the potential of photobiomodulation as an auxiliary tool in controlling orthodontic pain, demonstrating that the simplified protocol can be incorporated into clinical practice without significantly affecting appointment duration. Participants’ positive perception of the technique indicates a relevant subjective benefit, supporting treatment adherence and promoting a more comfortable experience.

However, the limited analgesic effects observed suggest the need for protocol refinement, considering factors such as application frequency, irradiation time, and number of treatment points. Future studies are essential to establish optimized standards that maximize the effectiveness of photobiomodulation in Orthodontics, making it an even more efficient and accessible tool in clinical practice.

## Data Availability

All data generated or analyzed during this study are included in this published article.
